# A new record of the southern erebid moth species, *Hypena nakajimai* Kishida, 2010 (Lepidoptera, Erebidae), from Korea with molecular analyses

**DOI:** 10.3897/BDJ.14.e198224

**Published:** 2026-06-19

**Authors:** Hee Han, Dong Ha Park, Sora Kim

**Affiliations:** 1 Department of Agricultural Convergence Technology, Jeonbuk National University, Jeonju, Republic of Korea Department of Agricultural Convergence Technology, Jeonbuk National University Jeonju Republic of Korea; 2 Lab. of Insect phylogenetics and evolution, Department of Bioenvironmental Chemistry, Jeonbuk National University, Jeonju, Republic of Korea Lab. of Insect phylogenetics and evolution, Department of Bioenvironmental Chemistry, Jeonbuk National University Jeonju Republic of Korea; 3 Department of Plastic and Reconstructive Surgery, Ajou University Hospital, Ajou University School of Medicine, Suwon, Republic of Korea Department of Plastic and Reconstructive Surgery, Ajou University Hospital, Ajou University School of Medicine Suwon Republic of Korea

**Keywords:** Erebidae, Hypeninae, *
Hypena
*, species report, taxonomy

## Abstract

**Background:**

The genus *Hypena* Schrank is a large but poorly-defined taxon. *Hypena nakajimai* Kishida, which is reported here for the first time in Korea, highlights the *Hypena*’s current taxonomic situation — a lack of phylogenetic analysis, taxonomic revision and numerous tropical species descriptions. *H. nakajimai* was initially found by Sugi more than 40 years ago, but the species was officially named by Kishida in 2010. It is expected that numerous undescribed and unknown *Hypena* species still remain to be discovered.

**New information:**

The present study reports the thirtieth Korean *Hypena* species, provides its diagnosis, re-describes its habitus and male genitalia and emphasises the importance of describing *Hypena* and its difficulties.

## Introduction

The genus *Hypena* Schrank is one of the speciose genera within the Lepidoptera (~ 500 species; Savela (2026)). The morphological characteristics of the genus *Hypena* were used to define its higher taxon, Hypeninae (Sisson et al. 2025). Adults bear a distinctive tuft protruding at the front of the head and labial palpi that are elongated with long second segments, which are the reasons why hypenine moths have been called “snouts” (Holloway 2008). Their larvae are typical semi-loopers without A3 prolegs (Holloway 2008). Additionally, there are some reports about economic importance from the *Hypena*, such as defoliation of hops (*Humulus lupulus* Linnaeus) by *Hypena humuli* Harris (Grasswitz and James 2008) and biocontrol of swallow-worts (*Vincetoxicum* Wolf) using *Hypena opulenta* Christoph (Hazlehurst et al. 2012).

The taxonomic history of *Hypena nakajimai* Kishida 2010 can be dated back to Inoue (1965), who first illustrated the moth with its Japanese vernacular name. Subsequently, Sugi (1982) recorded the species as “*Hypena* sp. 2” based on female samples. At that time, Sugi provided morphological diagnoses separating it from similar species, but the species remained without its formal scientific name for about 30 years. In 2007, male samples were successfully reared by S. Tominaga on Okinawa Island. These specimens were designated as the holotype and paratypes for the taxonomic description. The species was described as *Hypena nakajimai* sp. nov. by Kishida (2010).

In total, 29 *Hypena* moths have been recorded in Korea. Kononenko et al. (1998) reviewed 21 *Hypena* species and an unidentified *Hypena* species (*Hypena* sp. 8: Sugi (1982)). After that, Kononenko and Han (2007) reviewed 22 Korean *Hypena*, including *Hypena furva* Wileman, which had already been collected by Sugi (1982). Choi et al. (2015) reported *Hypena sinuosa* Wileman for the first time in Korea. Lee et al. (2022) recorded *Hypena narratalis* Walker, which is a species that hibernates in limestone caves (Kishida 2011). Recently, Jin et al. (2025) reported five *Hypena* from Korea in their checklist for Korean *Hypena*. Here, we add a species, *Hypena nakajimai* Kishida, to the list of *Hypena* in Korea.

## Materials and methods

### Taxon sampling

Living moth samples were killed with ammonia or ethyl acetate. All the collected samples were kept in a fridge. To make dried specimens, wings of samples were spread and we dried samples for about two to three weeks at 40°C in a drying oven. Before dissecting genitalia and DNA extraction, specimen photos were taken using the Canon EOS 6D (Canon IT Solutions Inc., Japan) and the Canon EF 100 mm F2.8L MACRO USM (Canon IT Solutions Inc., Japan). Multiple photos focused on specific regions of samples were stacked using the Helicon Focus software (version 8.2.2 Pro, Helicon Soft, Ukraine) for making a stacked image.

For taxonomic identification and writing descriptions, we dissected genitalia and made slide vouchers. First, the abdomen was separated from the specimen and heated overnight in a 10% solution of potassium hydroxide. The specific heating time is dependent on the size of the abdomen. The dissolved abdomen was descaled and cleaned using dissection tweezers and micro-brush pens in pure water and 70% ethyl alcohol, then stained with chlorazol black and mercurochrome. Considering the size of the abdomen, its time stained by chemicals can be fine-tuned. Its genitalia were isolated from the processed abdomen, soaked in 99% ethyl alcohol for dehydration and transferred to xylene for fixation. Both the genitalia and the abdomen, finally, were mounted on a slide glass with a moderate amount of Canada balsam. We took photos of genitalia using the Tucsen Dhyana 400 DC digital camera (Tucsen Photonics, China) and Leica S8AP0 stereomicroscope (Leica Microsystems, Germany). All of the samples and vouchers were examined using the EZ4 stereomicroscope (Leica Microsystems, Germany) and the naked eye.

### Molecular works

To compare the DNA information between *H. nakajimai* and *H. pulverulenta*, we performed DNA extraction, PCR amplification, sequencing and alignment for an *H. nakajimai* specimen. Genomic DNA was extracted from the specimen’s abdomen using the Omniprep Genomic DNA Extraction Kit (G-Biosciences, St. Louis, MO, USA), following Hamilton et al. (2019). The primer pair LEP-F1 (ATTCAACCAATCATAAAGATAT; Hebert et al. (2004)) and JHCO (CCTCTTTCTTGTGAAATAA; this study) were used to amplify a ~ 720-bp length of partial COI sequence, including the universal invertebrate barcode region. The thermal cycling programme comprised an initial denaturation stage at 95°C for 2 minutes, followed by 40 cycles of denaturation at 95°C for 30 seconds, annealing at 44.8°C for 30 seconds, extension at 72°C for 1 minute and a final extension at 72°C for 5 minutes. A chromatogram of a raw sequence was checked and edited using SeqMan^TM^ II (version 5.01, 2001; DNA-star^TM^). Although sequencing was attempted using both LEP-F1 and JHCO at Macrogen, Inc. (Seoul, Republic of Korea), the JHCO reverse reaction failed to generate a reliable read. Therefore, we only used the high-quality LEP-F1 forward read. The final 691-bp COI fragment, which was used for identification and phylogenetic analyses, was generated after trimming low-quality ends.

Phylogenetic analyses were conducted to check the phylogenetic relationship between *H. nakajimai* and *H. pulverulenta*. In total, 34 sequences for 19 species, including an outgroup species (*Paracolax tristalis*) and Korean *Hypena* species used in Jin et al. (2025), were used to perform phylogenetic analyses. MAFFT v.7.511 (Katoh et al. 2019) was employed to conduct multiple sequence alignments. On the one hand, the Maximum Likelihood (ML) approach was used to infer phylogenetic relationships between *Hypena* using IQ-TREE 2.4.0 (Minh et al. 2020). ModelFinder (Kalyaanamoorthy et al. 2017) was used to determine the best-fit substitution model. Nodal support was assessed using 1000 replicates of the Shimodaira-Hasegawa approximate likelihood-ratio test (SH-aLRT; Guindon et al. (2010)) and the most recent ultrafast bootstrap algorithm (UFBoot2; Hoang et al. (2018)) with nearest neighbour interchange (NNI) optimisation. On the other hand, the neighbour-joining (NJ) was generated using MEGA 11 (Tamura et al. 2021) with 1000 bootstrap replicates using the K2P model.

## Taxon treatments

### Hypena

Schrank, 1802

0B37C488-A798-5A34-8B56-938292ED1357

*Hypena* Schrank, 1802 - Schrank (1802) ; 163; TS: *Phalaena proboscidalis* Linnaeus, 1758 - Linnaeus (1758)
*Erichila* Billberg, 1820 - Billberg (1820); 89; TS: *Phalaena proboscidalis* Linnaeus, 1758- Linnaeus (1758)
*Herpyzon* Hübner, 1822 - Hübner (1822) ; 56; TS: *Phalaena proboscidalis* Linnaeus, 1758 - Linnaeus (1758)
*Bomolocha* Hübner, [1825] - Hübner (1816); 343; TS: *Phalaena crassalis* Fabricius, 1787 - Fabricius (1787)
*Ophiuche* Hübner, [1825] - Hübner (1816); 344; TS: *Phalaena lividalis* Hübner, 1796 - Hübner (1796)
*Badausa* Walker, [1863] - Walker (1863); 170; TS: *Badausa hypenoides* Walker, [1859] - Walker (1859)
*Peliala* Walker, 1865 - Walker (1865); 1005; TS: *Peliala tenebrosa* Walker, 1865 - Walker (1865)
*Phanaspa* Walker, [1866] - Walker (1866); 1192; TS: *Phanaspa dilatatalis* Walker, [1866] - Walker (1866)
*Plathypena* Grote, 1873 - Grote (1873a); 38; TS: *Hyblaea scabra* Fabricius, 1798 - Fabricius (1798)
*Euhypena* Grote, 1873 - Grote (1873a); 38; TS: *Hypena toreuta* Grote, 1872 - Grote (1872)
*Macrhypena* Grote, 1873 - Grote (1873a); 38; TS: *Hypena deceptalis* Walker, [1859] - Walker (1859)
*Lomanaltes* Grote, 1873 - Grote (1873b); 13; TS: *Lomanaltes laetulus* Grote, 1873 - Grote (1873b)
*Meghypena* Grote, 1873 - Grote (1873c); 86; TS: *Meghypena velifera* Grote, 1873d - Grote (1873c)
*Apanda* Moore, 1882 - Moore (1882); 186; TS: *Apanda denticulata* Moore, 1882 - Moore (1882)
*Mathura* Moore, 1882 - Moore (1882); 188; TS: *Mathura albisigna* Moore, 1882 - Moore (1882)
*Nesamiptis* Meyrick, 1899 - Meyrick (1899); 156; TS: *Nesamiptis plagiota* Meyrick, 1899 - Meyrick (1899)
*Ogoas* Druce, 1890, in Godman & Salvin, 1890 - Godman and Salvin (1881); 439; TS: *Ogoas albipuncta* Druce, 1890, in Godman & Salvin, 1890 - Godman and Salvin (1881)
*Tomyris* Druce, in Godman & Salvin, 1890 - Godman and Salvin (1881); 440; TS: *Tomyris nigropuncta* Druce, in Godman & Salvin, 1890 - Godman and Salvin (1881)
*Anepischetos* Smith, 1900 Smith (1900); 482; TS: *Anepischetos bipartita* Smith, 1900 Smith (1900)
*Trichypena* Joannis, 1915 - Joannis (1915); 6; TS: *Trichypena quadra* Joannis, 1915 - Joannis (1915)
*Rowdenia* Nye, 1975 - Nye (1975); 435; TS: *Tomyris nigropuncta* Druce, in Godman & Salvin, 1890 - Godman and Salvin (1881)
Hypena (Tetrastictypena) Lödl, 1994 - Lödl (1994a); 504; TS: *Hypena tetrasticta* Hampson, 1910 - Hampson (1910)
Hypena (Jussalypena) Lödl, 1994 - Lödl (1994a); 507; TS: Hypena (Jussalypena) jussalis Walker, [1859] - Walker (1859)
Hypena (Extremypena) Lödl, 1994 - Lödl (1994a); 553; TS: *Hypena extremipalpis* Lödl, 1994b - Lödl (1994b)
Hypena (Biangulypena) Lödl, 1994 - Lödl (1994a); 559; TS: *Hypena biangulatoides* Poole, 1989 - Poole (1989)
Hypena (Conscitalypena) Lödl, 1994 - Lödl (1994a); 564; TS: *Hypena conscitalis* Walker, [1866] - Walker (1866)
Hypena (Pseudodichromia) Lödl, 1994 - Lödl (1994a); 569; TS: *Hypena laetalis* Walker, [1859] - Walker (1859)
*Obesypena* Beck, 1996 - Beck (1996); 24; TS: *Hypena obesalis* Treitschke, 1829 - Treitschke (1829)
*Rostrypena* Beck, 1996 - Beck (1996); 24; TS: *Phalaena rostralis* Linnaeus, 1758 - Linnaeus (1758)

#### Diagnosis

This genus can be determined by the following characteristics: in habitus, male antennae ciliate; palpi extremely long; fore-wing elongated with acute apical angle; abdomen bearing tuft of scales on its first segment; in male genitalia, uncus short, with sharp apex; juxta plate-like shaped; valvae elongated, broadened, rounded; harpe absent; aedeagus curved; vesica bulbous; in female genitalia, both anterior and posterior apophyses the same in their length; ductus bursae thin and membranous; corpus bursae membranous, commonly as long as ductus bursae.

### Hypena nakajimai

Kishida, 2010

E182797F-B656-555E-95A0-39BAEB98298B

*Hypena nakajimai* Kishida 2010; 64; TL: Japan (Okinawa Pref., Okinawa I.)

#### Materials

- **Type status:** Other material. **Occurrence:** recordedBy: Dong Ha Park; individualCount: 1; sex: male; lifeStage: adult; occurrenceID: 479657F5-8079-5F87-8C06-3F22DE0D68B9; **Taxon:** scientificName: *Hypena nakajimai* Kishida, 2010; **Location:** continent: Asia; country: Korea; stateProvince: Jeollanam-do; county: Wando-gun; locality: Wando-eup, Jungdo-ri; **Identification:** identifiedBy: Hee Han; **Event:** eventDate: 08-10-2021; year: 2021; month: 10; day: 8; **Material Entity:** disposition: in the Jeonbuk National University collection

#### Description

Habitus (Fig. 1a, b). Male wingspan 19.1 mm. Ground colour yellowish-brown to orange-brown. Head scaled by normal tufts. Eyes large and globular. Antennae serrate, with yellow and brown scales. Proboscis well-developed. Labial palpi extremely long (approximately six times as long as the diameter of compound eye), tufted, covered with yellowish-brown or brown scales; second segment three to four times longer than third segment. Thorax covered by yellowish-brown or brown scales. Fore-wing ground colour yellowish-brown; entirely divided into two areas: dark costal and bright posterior area; a dark line arising from basal discal cell to the end of R5, which divides two areas mentioned above, posteriorly projected near the cell and forming V-shape opening towards costal margin; costal area usually dark brown or greyish-brown, but partially beige, especially at apex; posterior area usually yellowish-brown, but sometimes with dark scattered scales and partially brown or dark brown especially at middle area of subterminal and terminal fields (specifically, between R5 and M1 or M2); adterminal line dotted, innerly whitish, but distally dark brown or black; cilia dense, innerly blackish and distally brown or dark brown. Hind-wing uniformly pale yellowish-brown or greyish-brown, but innerly paler than outer area; cilia dense, pale yellowish-brown or dark brown. Upperside of both wings pale yellowish-brown or greyish-brown; in fore-wing, costal margin beige; apex dark brown and bearing a distinct spot, proximally black and distally whitish; between R4 and R5; in hind-wing, entirely faded yellowish-brown or beige, with scattered brownish scales at apex and costal area. Abdomen long, conical, yellowish or greyish-brown.

Male genitalia (Fig. 1c, d). Nearly symmetrical. Uncus strongly sclerotised, straight, weakly curved and sparsely setose at dorsal area, apically hooked, pointed and more sclerotised than other area. Subscaphium strongly developed as long as uncus; dorsally distinctively sclerotised and divided into two lobes. Tegumen two times longer than uncus, broad near uncus, but thin near vinculum. Vinculum slightly shorter than tegumen, innerly curved, but outerly straight, fused and broadened at downside. Saccus absent. Valvae tongue-like shaped, sparsely setose, proximal 3/4 area sclerotised, but distally membranous; costal area slightly bulbous at middle area; with a distinct division arising from basal valvae to middle area; and bearing a rounded saccular process at the middle of the division. Juxta plate inverted Y-shaped, with broad terminal branches and not fused with vinculum and valvae like its congener. Aedeagus entirely curved; coecum rounded; ductus ejaculatorius widely developed at dorsal area; carina dorsally strongly projected; vesica armed with a set of strong large cornuti and small spines.

#### Diagnosis

This species is extremely similar to *Hypena pulverulenta*. However, it can be distinguished by the following characteristics: in habitus, wingspan is shorter (~ 20 mm) than *H. pulverulenta* (~ 24 mm); fore-wing less elongated than in *H. pulverulenta*; in male genitalia, connected area between tegumen and vinculum more slender than in *H. pulverulenta*; valvae thinner and slightly more elongated than in *H. pulverulenta*; a distinct division at basal valvae more deeply divided than in *H. pulverulenta* and process arising from middle saccular area between the division much more notable than in *H. pulverulenta*; juxta diamond-shaped, but smaller than in *H. pulverulenta* and not fused with valvae and vinculum unlike *H. pulverulenta*; aedeagus strongly curved like *H. pulverulenta*, but thinner and more elongated than in *H. pulverulenta*; vesica armed with a set of strong cornuti, but *H. pulverulenta* not armed by them.

#### Distribution

Korea (new, JN), Japan (Shikoku, Kyushu, Amami Oshima, Okinoerabujima, Okinawa Island, Kohamajima and Ishigakijima).

#### Biology

*Commelina diffusa* Burman (Commelinaceae) is known as the host plant (Tominaga 2010).

#### Notes

This species is reported for the first time here in Korea. In diagnosis, while Sugi (1982) stated that *H. nakajimai* (*Hypena* sp. 2 in his book) could be distinguished by its fore-wing without dark scales, we found that the Korean specimen of *H. nakajimai* bears some dark scales on its fore-wings. Therefore, we decline the diagnostic key and carefully propose other morphological taxonomic keys by examining our material and published specimens (Sugi 1982, Kishida 2010, Jin et al. 2025). Notably, we firstly provide genital diagnostic keys for *H. nakajimai* and *H. pulverulenta*.

## Analysis

### Molecular analyses

The present study uses COI data from 34 species: 33 Hypeninae and one Herminiinae as an outgroup (Suppl. material 1). Both of the phylogenetic analyses, the ML and NJ results, show that *H. nakajimai* is sister to the remaining *Hypena*, including *H. pulverulenta* (Fig. 2). *H. pulverulenta* is clearly included in clade A (Fig. 2a) with high support (SH-aLRT = 97.4, UFBoot2 = 94 and NJ bootstrap support = 89; Fig. 2). The other topology of *Hypena* (Fig. 2) is mostly similar to the topology of Jin et al. (2025). Additionally, we found a sequence of *H. nakajimai* (accession number = PV203691), which is 100% the same as our novel sequence, from NCBI using NCBI BLAST (Johnson et al. 2008).

## Discussion

Here, we report *H. nakajimai* from Korea and provide its diagnosis, re-description, taxonomic comments and phylogenetic results. Due to a lack of comprehensive study of *Hypena*, *H. nakajimai* remained undescribed for approximately 30 years before receiving its scientific name. Considering its distinctive appearance and the presence of the type specimen, the time taken for describing it is significantly long. Although Lödl (1994a), Lödl (1994c), Lödl (1995), Lödl (1998a) and Lödl (1998b) deeply studied *Hypena* and provided profound insights about the genus, his studies were quite inclined to African taxa and there are still a lot of missing gaps in *Hypena* taxonomy and systematics. Those “gaps” are usually found in both the New and Old World tropics (Poole 1989) and *H. nakajimai*, a typical East Asian subtropical species, is a great example of such a poor taxonomic situation of *Hypena*. To increase the awareness of *Hypena*, it is strongly required of lepidopteran taxonomists to describe unknown species, compare similar taxa and even perform a comprehensive phylogenetic study or taxonomic revision.

## Supplementary Material

XML Treatment for Hypena

XML Treatment for Hypena nakajimai

4CCBC377-92D0-5137-B16F-CEFB2E977C78 10.3897/BDJ.14.e198224.suppl1 Supplementary material 1 Classification, taxon list and data source for the present study Data type genomic File: oo_1662500.xlsx https://binary.pensoft.net/file/1662500 Hee Han, Dong Ha Park, Sora Kim

## Figures and Tables

**Figure 1a.**
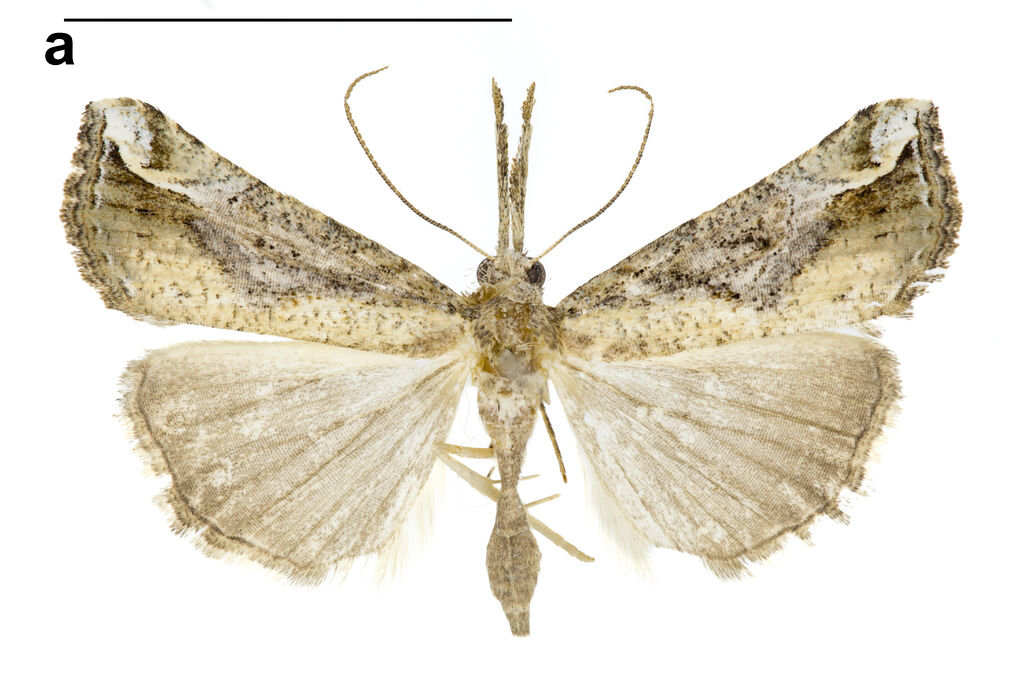
*Hypena nakajimai*, male, dorsal; scale bar: 10 mm;

**Figure 1b.**
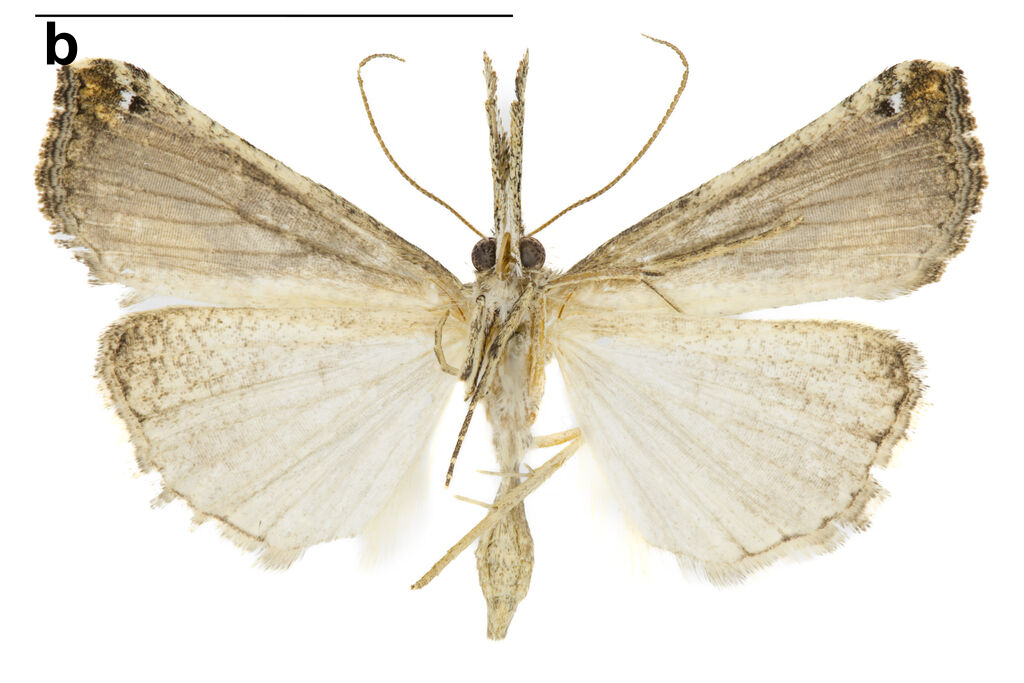
*Hypena nakajimai*, male, ventral; scale bar: 10 mm;

**Figure 1c.**
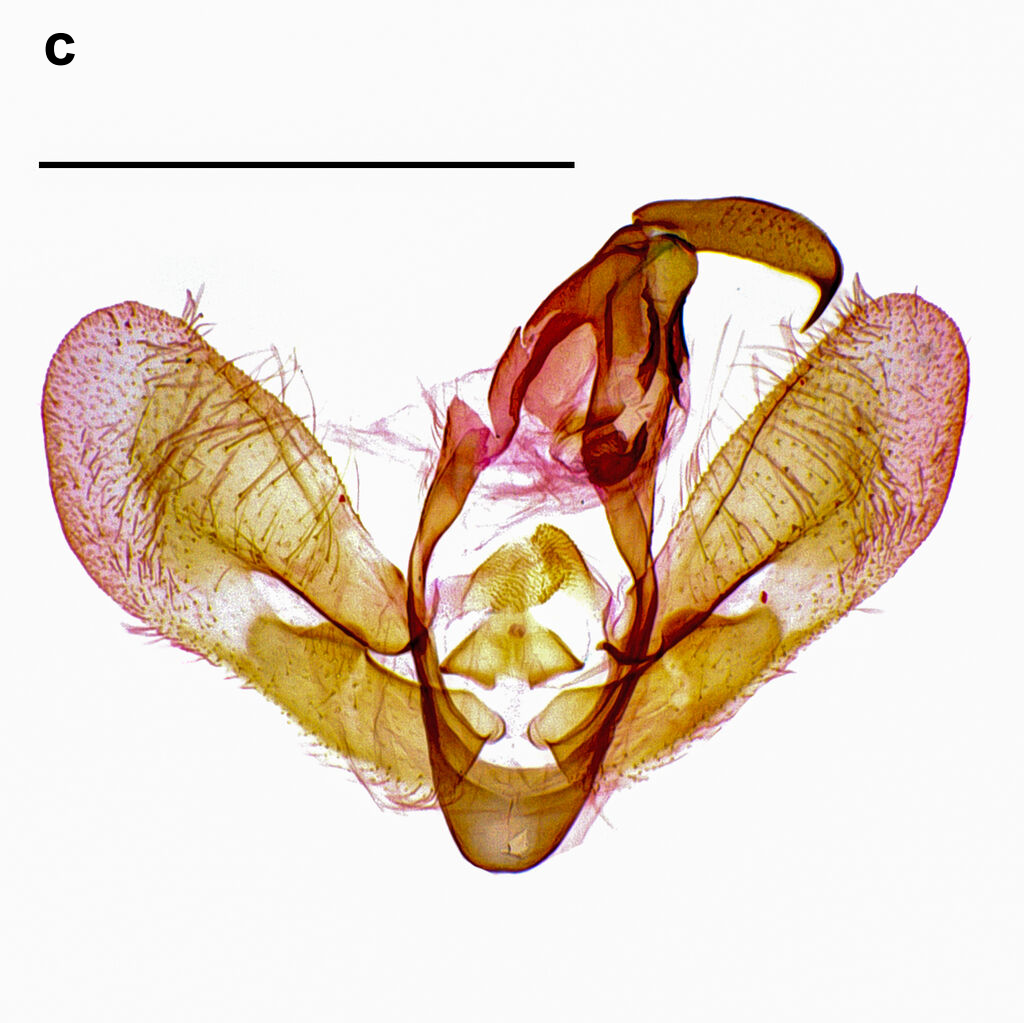
*Hypena nakajimai*, male genital capsule; scale bar: 5 mm;

**Figure 1d.**
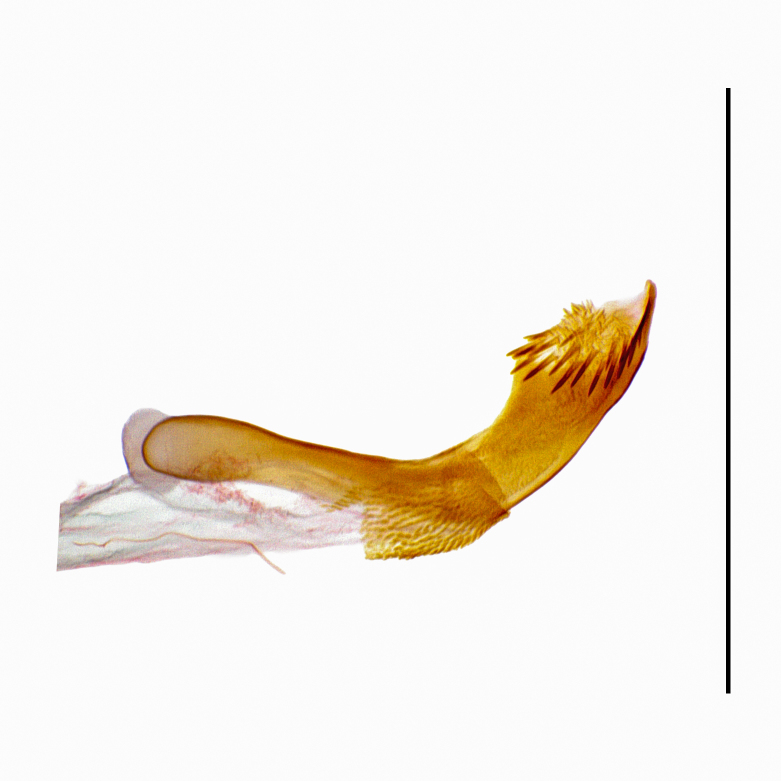
*Hypena nakajimai*, male aedeagus; scale bar: 5 mm.

**Figure 2a.**
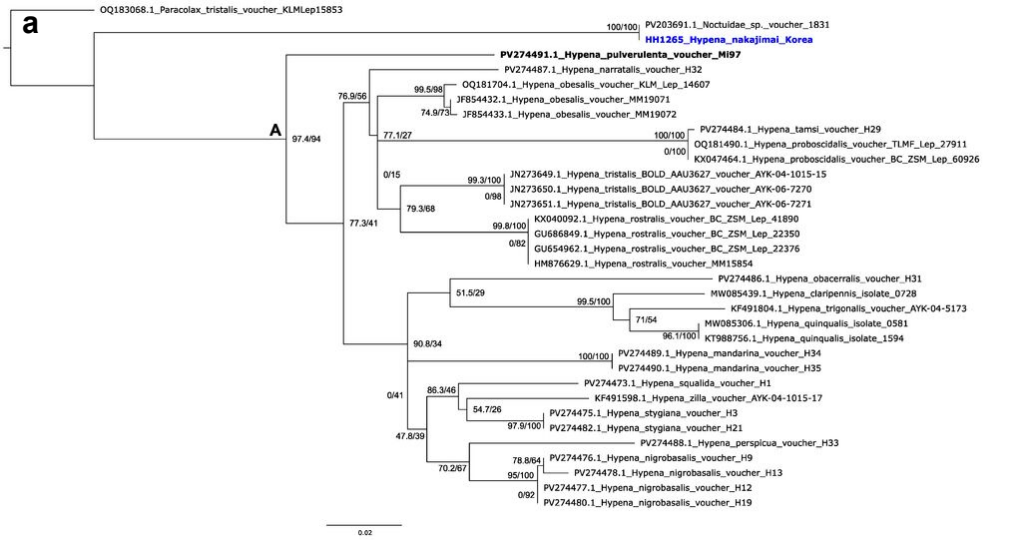
A Maximum Likelihood (ML) tree of *Hypena* and the phylogenetic position of *H. nakajimai*;

**Figure 2b.**
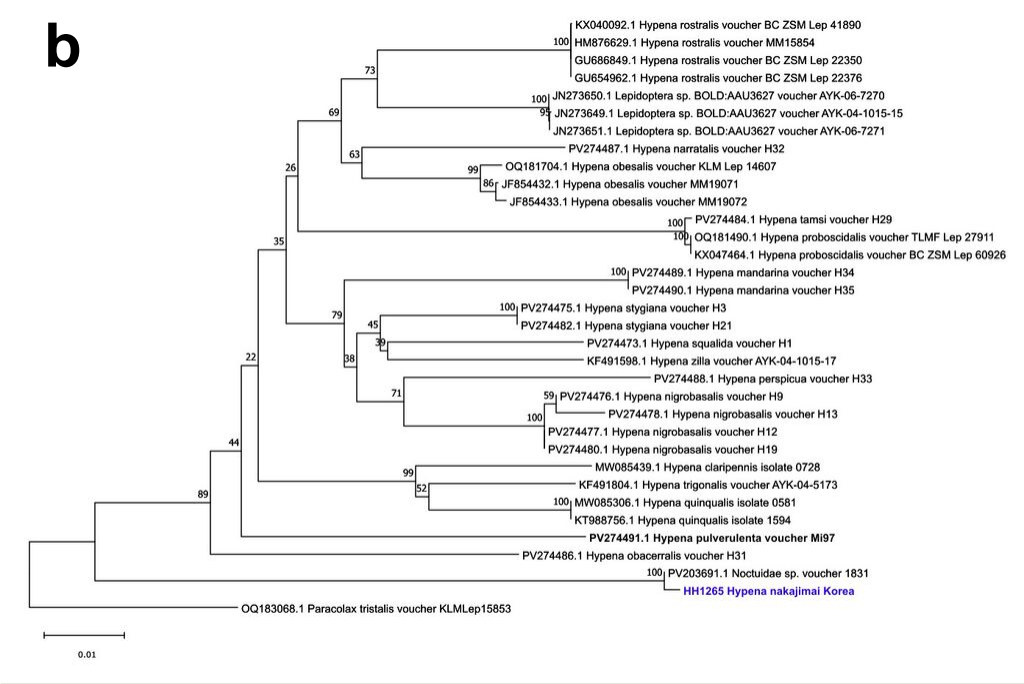
A neighbour-joining (NJ) tree of *Hypena* and the phylogenetic position of *H. nakajimai*.
